# Supplementary material for: CircTMTC1 contributes to nasopharyngeal carcinoma progression through targeting miR-495-MET-eIF4G1 translational regulation axis
Source: Cell Death Dis. 2022 Mar 18;13(3):250. doi: 10.1038/s41419-022-04686-z (PMC8930977; doi:10.1038/s41419-022-04686-z)
Supplement: Supplementary file 1 — Revised manuscript (marked-up) [file 41419_2022_4686_MOESM1_ESM.docx]

**CircTMTC1 contributes to** **nasopharyngeal carcinoma progression through targeting miR-495-MET-eIF4G1** **translational regulation axis**

**Running title:** CircTMTC1 contributes to NPC growth and metastasis

**Ya-Jie Zhao^1^, Chao Li^2^, Yan Zhang^3^, Zhan-Zhan Li^2,^***

^1^Department of Nuclear Medicine, Xiangya Hospital, Central South University, Changsha 410008, Hunan Province, P.R. China

^2^Department of Oncology, Xiangya Hospital, Central South University, Changsha 410008, Hunan Province, P.R. China

^3^Department of Pathology, the Second Affiliated Hospital of Zhengzhou University, Zhengzhou 450014, Henan Province, P.R. China

***Corresponding author: Dr. Zhan-Zhan Li,** Department of Oncology, Xiangya Hospital, Central South University, No.87, Xiangya Road, Changsha 410008, Hunan Province, P.R. China

**Email: zzhhaani789@163.com**

**Tel: +86-0731-89753332**

**Abstract**

Nasopharyngeal carcinoma (NPC) is the most common primary malignancy arising from the epithelial cells of nasopharynx. CircTMTC1 is upregulated in NPC patients, but its role and molecular mechanism in NPC is unknown. Normal nasopharyngeal epithelium and tumor tissues were collected. The expression of circTMTC1, miR-495, MET/eIF4G1 pathway-related molecules were examined. Colony formation and transwell assays were used to assess cell proliferation, migration and invasion. Cell apoptosis was analyzed by annexin V and propidium iodide (PI) staining. Gene interaction was examined by RNA immunoprecipitation (RIP) and luciferase activity assays. Subcutaneous and intravenous xenograft mouse models were established to analyze NPC growth and metastasis *in vivo*. CircTMTC1 was highly expressed and miR-495 was down-regulated in NPC, which were associated with poor prognosis of NPC. Both circTMTC1 knockdown and miR-495 overexpression inhibited NPC cell proliferation, migration, invasion and epithelial-mesenchymal transition (EMT) and promoted cell apoptosis. CircTMTC1 directly targeted miR-495 to promote the expression of its downstream target gene MET. miR-495 knockdown enhanced the expression of c-Myc, Cyclin D1 and survivin and accelerated NPC cell proliferation, migration, invasion and EMT through targeting MET and activating the MET-eIF4G1 axis. CircTMTC1 silence inhibited NPC growth and lung metastasis by targeting the miR-495-MET-eIF4G1 translational regulation axis *in vivo.* CircTMTC1 accelerates NPC progression through targeting miR-495 and consequently activating the MET-eIF4G1 translational regulation axis, suggesting potential therapeutic targets for NPC treatment.

**Keywords:** CircTMTC1, miR-495, MET-eIF4G1 axis, nasopharyngeal carcinoma

**Introduction**

NPC is the most common type of cancer in the nasopharynx^[1](#_ENREF_1)^, which is prevalent in some geographic areas including East and Southeast Asia and North Africa^[2](#_ENREF_2), [3](#_ENREF_3)^. Southern China has the highest incidence rate of NPC^[4](#_ENREF_4)^, which causes serious health burden. Due to the concealed localization and symptoms and its invasiveness, many NPC patients are with locally advanced cancer when they are first diagnosed, which also might be accompanied by distant metastasis^[5](#_ENREF_5)^. With the advance of radiotherapy and chemoradiotherapy for NPC patients, the prognosis has been greatly improved these years^[6](#_ENREF_6)^. However, for those patients with advanced cancer and distant metastasis, the therapeutic effect is still unsatisfactory^[7](#_ENREF_7)^. Therefore, elucidating underlying mechanisms is essential for identifying diagnostic and prognostic biomarkers and seeking novel therapeutic targets for NPC patients.

Circular RNAs (circRNAs) are evolutionarily conserved, covalently closed non-coding RNAs and ubiquitously expressed in mammalian cells^[8](#_ENREF_8)^, which exert important roles in regulating various physiological and pathological processes, such as cancers, by acting as a sponge for microRNAs (miRNAs) to reduce their abundance^[9-11](#_ENREF_9)^. Several circRNAs have been identified to be potential prognostic biomarkers and therapeutic targets in NPC^[12](#_ENREF_12)^. Hong et al. reported that circCRIM1 enhanced NPC metastasis and chemoresistance in NPC patients^[13](#_ENREF_13)^. Circ_0066755 promoted NPC cell proliferation and invasion by directly sponging miR-651, which could be used as a potential diagnostic biomarker^[14](#_ENREF_14)^. CircTMTC1 is identified as a novel circRNA with 458 nt length derived from TMTC1, but its biological functions are largely unknown. CircTMTC1 is significantly upregulated in NPC patients^[12](#_ENREF_12)^, suggesting that it might be involved in the regulation of NPC progression although there is still no evidence to prove it so far. Therefore, we aim to explore the role and underlying mechanism of circTMTC1 in NPC.

CircRNAs can work as miRNA sponges to reduce their expression, thereby relieving miRNA-mediated suppressive effects on down-stream gene targets^[15](#_ENREF_15)^. The circRNA-miRNA-mRNA regulatory network exerts important functions in various human cancers including NPC^[16](#_ENREF_16), [17](#_ENREF_17)^. miR-495 is involved in cancer cell proliferation, EMT and metastasis^[18](#_ENREF_18)^ and acts as a tumor suppressor in tumors, such as gastric cancer^[19](#_ENREF_19)^, endometrial cancer^[20](#_ENREF_20)^ and acute myeloid leukemia^[21](#_ENREF_21)^. Feng et al. proved that miR-495 was downregulated in radioresistant NPC tissues and sensitized NPC cell to radiotherapy through suppressing its EMT^[22](#_ENREF_22)^. However, the role of miR-495 and its regulation in NPC remain largely unknown.

The oncogene *MET* encodes a tyrosine kinase receptor and elicit an oncogenic activity in tumorigenesis^[23](#_ENREF_23), [24](#_ENREF_24)^. Increased expression of MET contributes to cancer cell proliferation, invasion and metastasis and is closely correlated with poor prognosis and radiotherapy resistance, which has been a prognostic biomarker and therapeutic target for various carcinomas^[25](#_ENREF_25)^. MET regulates HIF-1α expression via a translational mechanism dependent on the phosphorylation of eIF4G1 under hypoxia^[26](#_ENREF_26)^. Aberrant activation of MET pathway contributes to tumor progression by promoting tumor cell proliferation and EMT. For instance, MET promotes tumor cell proliferation via activating downstream targets including c-Myc^[27](#_ENREF_27)^. High expression of MET and Snail correlate with highly invasive tumor phenotypes in breast cancer^[28](#_ENREF_28)^. Importantly, MET is highly expressed in NPC tissues and its high expression correlates with shorter survival time of NPC patients^[29](#_ENREF_29)^, which could be targeted to regulate NPC cell growth and metastasis^[30](#_ENREF_30)^.

In summary, we hypothesized that circTMTC1 might regulate the progression of NPC through targeting miR-495 and activating the MET-eIF4G1 translational regulation axis. We examined whether circTMTC1 accelerated NPC cell proliferation, migration, invasion, EMT, metastasis and tumor growth. Our findings will clarify a novel regulatory mechanism of NPC progression and provide novel potential biomarkers and therapeutic targets.

**Results**

**Abnormal expression of circTMTC1 and miR-495 was associated with poor prognosis of NPC**

CircTMTC1 (circRNA ID: hsa_circ_0025767, chr12: 29,904,598–29,911,710), originating from exons 3, 4 and 5 of the *TMTC1* gene on the chromosome 12 (Fig. 1A), has been identified one of the top 20 circRNAs which are differentially expressed in NPC^[12](#_ENREF_12)^. The back-splicing site of circTMTC1 was validated by Sanger sequencing (Fig. 1A). Compared to TMTC1, circTMTC1 showed high resistance to RNase R digestion, indicating a stable circular structure of circTMTC1 (Fig. 1B). In addition, the half-time of circTMTC1 after actinomycin D treatment in 5-8F cells was much longer than that of TMTC1 mRNA (Fig. 1C), implying it was highly stable in NPC cells. Nuclear-cytoplasmic fractionation and fluorescence in situ hybridization (FISH) assays showed that circTMTC1 mainly located in the cytoplasm of 5-8F cells (Fig. 1D&E). We found that the circTMTC1 was markedly upregulated in NPC tissues (Fig. 1F), and circTMTC1^high^ patients showed obvious poor survival compared to circTMTC1^low^ patients (Fig. 1G). On the contrary, low expression of miR-495 was observed in NPC tissues, and miR-495^low^ NPC patients showed poor survival (Fig. 1H&I). Moreover, the expression of circTMTC1 was increased, but the expression of miR-495 was decreased in NPC cells, including 5-8F, C666-1, SUNE1 and 6-10B, compared to those in normal nasopharyngeal epithelial cell NP69 (Fig. 1J&K). 5-8F and SUNE1 cells showed highest expression of circTMTC1 and lowest expression of miR-495, which were selected for subsequent assays. These observations indicated that the abnormal expression of circTMTC1 and miR-495 in NPC patients predicted poor prognosis.

**CircTMTC1 knockdown and miR-495 overexpression suppressed NPC cell proliferation, migration and invasion and enhanced cell apoptosis**

To investigate whether circTMTC1 and miR-495 are involved in the regulation of NPC progression, we modified their expression by sh-circTMTC1 or miR-495 mimics transfection in NPC cells. CircTMTC1 and miR-495 were efficiently knocked down or overexpressed in NPC cells by sh-circTMTC1 or miR-495 mimics transfection respectively (Fig. 2A&B). Both circTMTC1 knockdown and miR-495 overexpression significantly inhibited colony formation in NPC cells (Fig. 2C&D). Enhanced cell apoptosis was observed in circTMTC1-knockdown or miR-495-overexpressing cells (Fig. 2E&F). Moreover, circTMTC1 knockdown and miR-495 overexpression also obviously reduced NPC cell migration and invasion (Fig. 2G-J&Supplementary Fig. 1A&B). Next, we examined the expression of EMT related factors E/N-cadherin^[31](#_ENREF_31)^, Snail^[32](#_ENREF_32)^, and proliferation related factors c-Myc^[33](#_ENREF_33)^, Cyclin D1^[34](#_ENREF_34)^ and Survivin^[35](#_ENREF_35)^. These results showed that both circTMTC1 knockdown and miR-495 overexpression upregulated E-cadherin but downregulated N-cadherin, Snail, c-Myc, Cyclin D1 and Survivin in NPC cells (Fig. 2K&L). To be concluded, circTMTC1 knockdown and miR-495 overexpression inhibited NPC cell proliferation, migration, invasion and EMT and enhanced cell apoptosis.

**CircTMTC1 directly bound miR-495 to reduce its abundance in NPC**

To explore the interaction between circTMTC1 and miR-495, we analyzed and found that the expression of circTMTC1 and miR-495 were negatively correlated in NPC tissues (Fig. 3A). Moreover, the abundance of miR-495 was increased in circTMTC1-knockdown 5-8F and SUNE1 cells (Fig. 3B), indicating that circTMTC1 suppressed miR-495 expression in NPC. To explore whether circTMTC1 directly bound to miR-495, we predicted and mapped a binding site for miR-495 in circTMTC1 using CircInteractome^[36](#_ENREF_36)^ (https://circinteractome.nia.nih.gov/, Fig. 3C). Luciferase assay showed that miR-495 overexpression obviously inhibited the luciferase activity of the wildtype circTMTC1 reporter but not the mutated one in NPC cells (Fig. 3D). Moreover, both circTMTC1 and miR-495 were enriched in the [immunoprecipitate](javascript:;)d fractions by anti-Ago2 (Fig. 3E). miR-495 could be enriched by the circTMTC1 probe in 5-8F and SUNE1 cells (Fig. 3F). Moreover, the localization of circTMTC1 and miR-495 was largely merged in the cytoplasm of NPC cells (Fig. 3G). To conclude, circTMTC1 targeted miR-495 to decrease its expression in NPC.

**CircTMTC1 contributed to NPC progression via targeting miR-495**

To demonstrate whether circTMTC1-mediated regulation of NPC was dependent on miR-495, circTMTC1 and miR-495 were overexpressed simultaneously in 5-8F and SUNE1 cells. The expression of circTMTC1 was markedly enhanced by circTMTC1 transfection (Fig. 4A). Compared to vector control, circTMTC1 overexpression promoted colony formation in NPC cells, which was abolished by simultaneous miR-495 overexpression (Fig. 4B&C). CircTMTC1-mediated suppressive effect on cell apoptosis was also abrogated by miR-495 overexpression (Fig. 4D&E). Additionally, miR-495 overexpression significantly suppressed the accelerative effects of circTMTC1 overexpression on NPC cell migration and invasion (Fig. 4F-I&Supplementary Fig. 1C&D). Increased expression of N-cadherin, Snail, c-Myc, Cyclin D1 and Survivin and decreased E-cadherin expression in circTMTC1-overexpressing 5-8F and SUNE1 cells were fully reversed by simultaneous miR-495 overexpression (Fig. 4J&K). Taken together, these observations suggested that circTMTC1 promoted NPC cell malignant phenotypes and suppressed cell apoptosis through directly targeting miR-495.

**The MET-eIF4G1 translational control axis was a downstream target of miR-495 and circTMTC1 in NPC**

Next, we predicted MET as a putative downstream target of miR-495. Therefore, we analyzed the expression of MET and its correlation with circTMTC1 and miR-495 in NPC patients. The results showed that MET expression was increased in NPC, and MET^high^ patients showed poor survival (Fig. 5A&B). Moreover, MET expression positively correlated with the expression of circTMTC1 but negatively correlated with miR-495 expression in NPC patients (Fig. 5C&D). A putative binding site for miR-495 in MET was predicted using Starbase^[37](#_ENREF_37)^ (http://starbase.sysu.edu.cn/index.php, Fig. 5E). To confirm the direct interaction between miR-495 and MET, the wildtype or mutated binding site for miR-495 in the 3'-UTR of MET was constructed into a luciferase reporter. miR-495 overexpression significantly impaired the luciferase activity of the wildtype MET reporter but not the mutated one in NPC cells (Fig. 5F), suggesting that miR-495 directly targeted MET in NPC. Compared to sh-NC and mimics NC, sh-circTMTC1 and miR-495 mimics transfection inhibited the expression of MET and its phosphorylation (Fig. 5G-I). Moreover, the expression and phosphorylation of eIF4G1, which has been identified as a target of MET to regulate protein translation^[26](#_ENREF_26)^, were also suppressed by circTMTC1 knockdown or miR-495 overexpression in NPC cells (Fig. 5H&I). These data demonstrated that circTMTC1 might target miR-495, thereby regulating the MET-eIF4G1 translational control axis in NPC.

**miR-495 knockdown promoted NPC progression by activating the MET-eIF4G1 axis**

To investigate whether miR-495-mediated regulation of NPC was dependent on the MET-eIF4G1 axis, miR-495 was knocked down in 5-8F and SUNE1 cells by miR-495 inhibitor transfection (Fig. 6A). Besides, MET was silenced via sh-MET transfection (Fig. 6B&C). miR-495 knockdown increased colony formation (Fig. 6D&E), inhibited cell apoptosis (Fig. 6F&G) and promoted cell migration (Fig. 6H&I) and invasion (Fig. 6J&K) in NPC cells. However, these effects were all fully abolished by simultaneous MET knockdown (Fig. 6D-K). In addition, 4EGI-1, an inhibitor of eIF4E/eIF4G interaction^[38](#_ENREF_38)^, could also abrogated all these miR-495 knockdown-mediated effects on NPC cell proliferation, apoptosis, migration and invasion (Fig. 6D-K&Supplementary Fig. 1E&F). Compared to inhibitor NC control, miR-495 silence inhibited E-cadherin expression but upregulated N-cadherin, Snail, c-Myc, Cyclin D1 and Survivin in NPC cells (Fig. 7A&B). The expression levels of these factors after miR-495 inhibitor transfection were partially rescued by simultaneous MET knockdown or 4EGI-1 treatment (Fig. 7A&B). Taken together, miR-495 silence promoted NPC cell malignant phenotypes and suppressed cell apoptosis by activating the MET-eIF4G1 translational regulation axis.

**CircTMTC1 silence inhibited NPC growth and lung metastasis via targeting the miR-495-MET-eIF4G1 axis *in vivo***

To explore whether circTMTC1 regulates NPC growth and metastasis *in vivo*, subcutaneous and intravenous mouse models were established as previously described^[39](#_ENREF_39)^. NPC cells with stable knockdown of circTMTC1 were subcutaneously grafted into BALB/c nude mice. Tumor volume and weight were dramatically inhibited in mice inoculated with circTMTC1-knockdown NPC cells (Fig. 8A-C). As expected, circTMTC1 silence reduced circTMTC1 expression and MET mRNA level and increased miR-495 expression in subcutaneous tumor tissues (Fig. 8D). Immunohistochemistry (IHC) staining showed that the expression of Ki-67, MET and eIF4G1 was much lower in circTMTC1 silence tumor tissues than that in sh-NC control tumor tissues (Fig. 8E). CircTMTC1 knockdown enhanced E-cadherin expression but suppressed the expression of N-cadherin, MET, eIF4G1, Snail, c-Myc, Cyclin D1 and Survivin and the phosphorylation of MET and eIF4G1 (Fig. 8F&G), demonstrating that circTMTC1 promoted NPC growth, proliferation and EMT by regulating the miR-495-MET-eIF4G1 axis. Furthermore, we investigated whether circTMTC1 regulated NPC cell metastasis by intravenously injecting 5-8F and SUNE1 cells into nude mice. CircTMTC1 silence obviously reduce the number of tumor nodules in the lung, which was also confirmed by histopathological staining (Fig. 8H&J), suggesting that circTMTC1 accelerate NPC metastasis *in vivo*. Taken together, circTMTC1 contributed to NPC growth and metastasis by targeting the miR-495-MET-eIF4G1 axis *in vivo*.

**Discussion**

NPC is the most common head and neck carcinoma and shows distinctly geographical distribution, which might be associated with Epstein-Barr virus infection and environmental factors^[40](#_ENREF_40), [41](#_ENREF_41)^. Although curative effects have improved owing to advanced radiotherapy and chemotherapy, the prognosis of patients with advanced cancer is still poor. Distant metastasis is the major barrier and the primary cause of NPC-related death^[42](#_ENREF_42)^. Therefore, exploring the regulatory mechanisms of NPC cell growth and metastasis is of crucial for developing novel therapeutic strategies. In this study, we found that circTMTC1 was upregulated and miR-495 was downregulated in NPC, which correlated with poor prognosis. CircTMTC1 enhanced the malignant phenotypes of NPC cells and NPC progression via targeting the miR-495-MET-eIF4G1 axis. Our study sheds light on the molecular mechanism of NPC progression and contributes to developing novel therapeutic management of NPC.

CircRNAs play key roles in regulating NPC growth and metastasis by acting as miRNA sponges. Yin et al. reported that hsa_circ_0046263 was up-regulated in NPC and accelerated NPC growth and metastasis by acting as a miR-133a-5p sponge^[43](#_ENREF_43)^. NPC cell proliferation and invasion were promoted by circSERPINA3 through targeting the miR-944/MDM2 axis^[44](#_ENREF_44)^. However, the role of circTMTC1 in cancers including NPC has not been reported yet. As previously reported^[12](#_ENREF_12)^, we found high expression of circTMTC1 in NPC cells and tissues and patients with its high expression had poor survival for the first time. Furthermore, we firstly demonstrated that circTMTC1 promoted cell proliferation, invasion and metastasis and suppressed cell apoptosis in NPC, identifying a novel role of circTMTC1 in regulating NPC progression.

Emerging evidence indicates circRNAs and long non-coding RNAs (lncRNAs) function as sponges for miRNAs to relieve miRNA-mediated regulatory effects on downstream genes, described as a competing endogenous RNA (ceRNA) regulatory network^[45](#_ENREF_45), [46](#_ENREF_46)^, which plays key roles in regulating tumor progression^[47](#_ENREF_47)^. Li et al. found that circTGFBR2 functioned as a ceRNA to inhibit NPC progression via sponging miR-107^[48](#_ENREF_48)^. CircZNF609 accelerates NPC growth and metastasis via competing with miR-150-5p^[49](#_ENREF_49)^. In this study, we firstly demonstrated that circTMTC1 contained a binding site of miR-495 and targeted miR-495 to promote NPC growth and metastasis. miR-495 acts as an important tumor suppressor in cancers. Yan et al. reported that miR-495 reduced colorectal cancer cell proliferation and migration^[50](#_ENREF_50)^. Liu and colleagues reported that miR-495 restrained EMT and metastasis of gastric cancer cells^[51](#_ENREF_51)^. However, whether miR-495 exerts anti-tumor activity to suppress NPC cell proliferation, migration and metastasis is unknown. As reported previously in various human cancers^[52](#_ENREF_52), [53](#_ENREF_53)^, we also found that miR-495 could elicit strong anti-tumor activity in NPC. miRNAs generally play their roles via directly binding to target mRNAs^[54](#_ENREF_54)^. Several targets of miR-495 have identified, such as FAM83D^[50](#_ENREF_50)^, HOXC6^[55](#_ENREF_55)^ and PBX3 and MEIS1^[21](#_ENREF_21)^. Intriguingly, we identified that MET was a novel target of miR-495 and miR-495-mediatd effects in NPC was dependent on MET. However, as a miRNA generally has many target genes, whether other targets, such as FAM83D, HOXC6 and PBX3 and MEIS1 we mentioned, are involved in miR-495-mediated anti-tumor activity in NPC is still needed to be clarified.

MET provides essential signals for cell proliferation, survival and migration, which can be hijacked by carcinoma cells for growth and metastasis^[56](#_ENREF_56)^. Therefore, MET has emerged as a key target for tumor therapy. In NPC, MET overexpression was obviously implicated in metastasis and poor survival of patients^[57](#_ENREF_57)^. Moreover, Li et al. reported that MET knockdown inhibited NPC cell proliferation, migration and invasion^[30](#_ENREF_30)^. The expression of MET and phosphorylated MET has been studied in cancers^[58](#_ENREF_58)^. Phosphorylation of MET enhanced its tyrosine kinase activity, which leads to autophosphorylation or phosphorylation of downstream targets including eIF4G1^[26](#_ENREF_26), [59](#_ENREF_59)^. Aberrant activation of MET occurs in human cancers and is regulated through various mechanisms, such as miRNA-mediated regulation. Here, we found that knockdown of circTMTC1 or overexpression of miR-495 increased the phosphorylation of MET in NPC cells. Intriguingly, we demonstrated that MET was directly targeted and regulated by miR-495 and identified a novel circTMTC1-miR-495-MET ceRNA network in NPC.

Cancer cells require highly controlled protein translation to maintain the expression of oncogenes. Protein translation is tightly regulated by different components of EIF4F complex^[60](#_ENREF_60)^. EIF4G and EIF4E are important for the EIF4F complex and cap-dependent protein translation^[61](#_ENREF_61)^. MET phosphorylation leads to subsequent ERK1/2-mediated phosphorylation of eIF4G1 on Ser-1232, and the MET-eIF4G1 axis was identified as a translational regulation axis under hypoxia^[26](#_ENREF_26)^. In addition, activation of MET regulates E-cadherin and vimentin to induce EMT and promotes cancer cell proliferation through activating downstream targets such as c-Myc^[27](#_ENREF_27), [62](#_ENREF_62)^. In this study, high MET expression in NPC indicated aberrant activation of MET-eIF4G1 axis. Knockdown of MET reversed miR-495 inhibitor-mediated regulation of E-cadherin, N-cadherin, Snail, c-Myc, Cyclin D1 and surviving. Importantly, we found that eIF4G1 expression and its phosphorylation were suppressed by circTMTC1 knockdown and blocking the MET-eIF4G1 axis abolished miR-495 silence-mediated effects on NPC cells. To summarize, we demonstrated that circTMTC1 promoted the expression and phosphorylation of MET and thus activated eIF4G1 and downstream proliferation-related signaling via targeting miR-495, thereby accelerating NPC cell proliferation and metastasis.

To conclude, we firstly demonstrate that circTMTC1 contributes to NPC cell proliferation, migration, invasion and metastasis and accelerates NPC progression by targeting miR-495, consequently increasing the expression and phosphorylation of MET and eventually activating eIF4G1 signaling. Our study not only elucidates a novel regulatory mechanism of NPC progression, but also identifies potential prognostic biomarkers and therapeutic targets for NPC. To achieve this, more investigations are ongoing to elucidate the nature of the regulation in detail.

**Materials and Methods**

**Patient specimens**

We collected thirty-two NPC tissues and sixteen nasopharyngeal epithelial tissues from patients with NPC or chronic nasopharyngeal inflammation at the Xiangya Hospital of Central South University. The patients received none preoperative treatments prior to the sample collection. Samples were stored at -80°C for analyzing the expression of circTMTC1, miR-495 and MET. Overall survival rate of patients was monitored for 60 months. This study got approval from the Ethics Committee of the Xiangya Hospital of Central South University. All patients provided written informed consent.

**Cell culture**

Human NPC cells 5-8F, C666-1, SUNE1 and 6-10B and normal nasopharyngeal epithelial cell NP69 were purchased from Chinese Academy of Sciences Cell Bank (Shanghai, China) and kept in the Dulbecco's Modified Eagle's medium (DMEM, ThermoFisher Scientific, Waltham, MA, USA) containing 10% fetal bovine serum (FBS, ThermoFisher Scientific). Cells were tested without contamination with mycoplasma. Medium was replaced every day. Passage 4-12 cells were used for subsequent assays.

**Cell transfection**

miR-495 mimics/inhibitor, mimics/inhibitor NC, shRNA against circTMTC1 and MET and shRNA NC were all bought from RiboBio (Guangzhou, China). For stable transfection, sh-circTMTC1 was cloned into the pGFP-C-shLenti vector, and lentiviral particles were packaged in HEK293T cells for knockdown of sh-circTMTC1 in NPC cells. Puromycin (5 μg/mL, Sigma-Aldrich, St. Louis, MO, USA) was supplemented in the medium for 1 week for screening stable cell clones after lentiviral infection. CircTMTC1 was inserted into the pcDNA3.1(+) CircRNA Mini Vector from Addgene (Watertown, MA, USA) for its overexpression. For transient transfection, NPC cells were transfected with sh-NC, sh-circTMTC1, mimics NC, miR-495 mimics, inhibitor NC, miR-495 inhibitor or sh-MET using Lipo 3000 cell transfection reagent (ThermoFisher Scientific) respectively following the manual. 48 h later, cells were collected for subsequent assays.

**Nuclear-cytoplasmic fractionation**

NE-PER Nuclear and Cytoplasmic Extraction Reagents obtained from Thermo Fisher Scientific were used for nuclear-cytoplasmic fractionation. Cells were trypsinized and harvested. 2×10^6^ cells were transferred into a new tube and pelleted. Subsequently, the supernatant was discarded, and ice-cold CER I solution was added into the pellet prior to separation of nuclear and cytoplasmic fractions following the manual.

**FISH assay**

NPC cells were fixed in 4% formaldehyde solution for 20 min, washed twice in PBS and dehydrated in gradient ethanol solution (50%, 75%, 95% and 100%). Cells were hybridized at 56 °C for half an hour with Alexa Fluor 555-conjugated oligonucleotide probe against miR-495 or Alexa Fluor 488-conjugated oligonucleotide probe against circTMTC1 at 20 nM. Probes were purchased from Genepharma (Shanghai, China). Next, cells were rinsed and stained with DAPI (Abcam, Cambridge, UK) for 10 minutes in dark. Cells were then mounted in ProLong Gold Antifade Mountant (ThermoFisher Scientific). Slides were imaged with a Leica confocal system (Weztlar, Germany).

**CircTMTC1** **characterization analysis**

For analyzing the resistance of circTMTC1 to RNase R digestion, total RNA was extracted and digested with RNase R (BioVision, Milpitas, CA, USA) at 2 U/μg for 1 hour at 37 °C. CircTMTC1 was then quantified with qRT-PCR. For analyzing the half-time of circTMTC1, the gene transcription was inhibited by adding 2 μg/mL of Actinomycin D (Sigma-Aldrich) into the culture medium. The half-time of circTMTC1 and TMTC1 was examined using qRT-PCR. PCR products amplified with the divergent primer were used for Sanger sequencing to confirm the back-splicing site of circTMTC1.

**Colony formation analysis**

NPC cells with indicated transfection were seeded each well in 6-well plates and incubated in DMEM in a cell incubator for 2 weeks. The medium was replaced every 3 days. After 2 weeks, cell colonies were rinsed and fixed in 4% formaldehyde solution. After wash in PBS, cells were then stained with crystal violet solution (Sigma-Aldrich). Cell colonies were then imaged with a BX51 microscope (Olympus, Tokyo, Japan) and quantified using ImageJ software.

**Cell apoptosis analysis**

Cell apoptosis was examined with Annexin V Apoptosis Detection Kit (BioLegend, San Diego, CA, USA) following the manual. Briefly, 1×10^5^ NPC cells with indicated transfection were rinsed in PBS and incubated in 100µL of binding buffer with 5 µL of annexin V-FITC and 10 µL of PI for 15 min. Next, 400 µL of binding buffer was added, and cells were immediately analyzed with a flow cytometer from BD Biosciences (Franklin Lakes, NJ, USA).

**Scratch wound healing assay**

Cell migration was evaluated with the scratch wound healing assay. 5-8F and SUNE1 cells were grown to a confluent monolayer. Culture medium was removed, and the cell monolayer was scratched by drawing across with the cell comb from EMD Millipore (Darmstadt, Germany). Subsequently, cells were cultured for additional 24 h for wound healing. Finally, the healing was observed under a BX51 microscope (Olympus) and quantified with the image J software.

**Transwell assays for migration and invasion**

Transwell chambers with 8 µm pore from Corning (Corning, NY, USA) were used for examining NPC cell migration and invasion. For cell migration analysis, 1×10^5^ NPC cells with indicated transfection were washed and plated into the upper chamber. DMEM containing 10% FBS was added into the lower chamber, cells were incubated in a cell incubator for 12 h. For cell invasion analysis, a similar assay was performed except that the upper chamber was pre-coated with Matrigel (BD, Franklin Lakes, NJ, USA) and incubated for 24 h. Next, the migratory and invasive cells in the lower chamber were washed, fixed and stained with crystal violet solution (Sigma-Aldrich), which were then imaged with a BX51 microscope (Olympus, Tokyo, Japan).

**Dual-luciferase reporter assay**

Wildtype (WT) and mutated (MUT) binding sites of miR-495 in circTMTC1 and the 3' untranslated region (3'-UTR) of MET were constructed into pmirGLO vectors (Promega, Madison, WI, USA). NPC cells were co-transfected with circTMTC1 or MET reporter and miR-495 mimics. Mimics NC was used as a control. After 48 h, cells were harvested, and the luciferase activity was examined with Dual-Glo Luciferase Assay System (Promega).

**RIP**

NPC cells were grown to 90% confluency and washed twice in PBS. Cells were lysed in lysis buffer supplemented with ribonuclease and protease inhibitors for half an hour on ice. The supernatants of cell lysates were harvested after centrifugation. 10 µL of supernatants were aliquoted and used as input. Protein magnetic beads were pre-coated with a rabbit Ago-2 antibody (ab186733, Abcam), which were added into lysates and incubated with gentle rotation at 4 °C overnight. Normal rabbit IgG was used as a control. Subsequently, RNA was recovered and quantified using quantitative reverse transcription polymerase chain reaction (qRT-PCR). Results were shown as the relative enrichment to input.

**RNA pull-down assay**

The RNA-Protein pull-down kit was provided by Thermo Fisher Scientific, and RNA pull-down assays were performed following the manual. Briefly, cells were lysed, and the supernatants were collected. Subsequently, the supernatants were mixed with biotin-labeled circTMTC1 probes and incubated for 6 h. Streptavidin-magnetic beads were added, and samples were incubated for 2 h. Finally, RNA was eluted and examined by qRT-PCR.

**NPC growth and metastasis mouse models**

BALB/c nude mice (6-week old, male) were bought from SJA Laboratory Animal Co., Ltd (Hunan, China; n=32), and were randomly divided into 4 groups with 8 mice in each group. The investigator was blinded to the group allocation during the experiment. For NPC growth mouse model, 1×10^6^ 5-8F and SUNE1 cells with stable knockdown of circTMTC1 were suspended and subcutaneously injected into the right flanks of mice. Tumor size was monitored every 5 days up to 35 days and the volume was calculated with the formula length×width^2^/2. Mice were sacrificed after 5 weeks, and tumor tissues were excised and weighed for subsequent assays. For lung metastasis analysis, 1×10^6^ aforementioned 5-8F and SUNE1 cells were suspended in 100 µL of PBS and injected to mice via the tail vein. After five weeks, mice were sacrificed, and the lungs were excised for photographing and hematoxylin and eosin staining. The metastatic nodules were quantified. Mice will be excluded if they lose their appetite completely for 24 h or have poor appetite (less than 50% of the normal amount) for 3 days. Animal experiments were approved by the Animal Care and Use Committee of the Xiangya Hospital of Central South University.

**Hematoxylin and eosin (H&E) staining**

The mice lung tissues were then fixed in 4% formaldehyde solution overnight. Second day, the lungs were dehydrated, embedded in paraffin and sliced into 5-µm sections. Sections were deparaffinized twice in xylene for 10 min and rehydrated in gradient ethanol twice for 10 min in each gradient ethanol (100%, 95%, 70% and 50%) followed by immersion in deionized water twice. Sections were stained with hematoxylin for 3 min, washed thoroughly, differentiated in 0.3% acid alcohol and stained with eosin for 2 min. Sections were then washed and cleared in xylene. Sections were mounted and imaged using a BX51 microscope (Olympus).

**IHC staining**

Tumor tissues were fixed in 4% formaldehyde solution, dehydrated in gradient ethanol solution, embedded in paraffin and cut into 5-µm sections. After antigen retrieval, sections were incubated in H_2_O_2_ solution for 10 min, washed and incubated with primary antibodies against Ki-67 (1:100, ab16667, Abcam), MET (1:200, ab51067, Abcam) and eIF4G1 (1:100, ab2609, Abcam) for 16 h. Sections were then incubated with HRP-conjugated secondary antibody (1:1000, ThermoFisher Scientific). DAB substrate was added to visualize the signal. After wash, sections were stained with hematoxylin and imaged using a BX51 microscope (Olympus).

**RNA extraction and qRT-PCR**

Total RNA was extracted from NPC tissues, normal nasopharyngeal epithelium tissues, subcutaneous xenograft tumors, NPC cells with TRIzol reagent (ThermoFisher Scientific). RNA was quantified using a NanoDrop 2000 spectrophotometer (ThermoFisher Scientific). For circRNAs, DNA was digested using DNase I and ribosomal RNAs were removed. RNase R treatment was used to enrich circRNAs. For miRNAs, miRNAs were isolated with miRcute miRNA Isolation Kit (DP501, TIANGEN, Beijing, China). Then, circRNA, mRNA and miRNA were reversely transcribed into cDNA respectively. The relative expression of circTMTC1, miR-495 and MET were examined by quantitative PCR using Power SYBR Green PCR Master Mix (Thermo Fisher Scientific). CircTMTC1 and MET was normalized to GAPDH. miR-495 was normalized to U6 snRNA. Results were calculated with the 2^−∆∆Ct^ method. Primers used here were listed in Table 1.

**Western blotting**

NPC cells with indicated transfection were lysed in radio-immunoprecipitation assay (RIPA) lysis buffer (Santa Cruz, Dallas, TX, USA) for half an hour on ice. Excised xenograft tumors were homogenized and lysed in RIPA lysis buffer (Santa Cruz) for 1 hon ice. The supernatants were harvested after centrifugation at 10,000 g for 15 min. Protein was quantified with BCA assay kit (ThermoFisher Scientific). 30 μg of protein was loaded, electrophoresed and transferred to polyvinylidene fluoride (PVDF) membrane (Bio-Rad, Hercules, CA, USA). Membranes were then blocked in 5% bovine serum albumin (BSA) solution for 1 h. After wash, membranes were incubated with primary antibodies against E-cadherin (1:1000, ab231303, Abcam), N-cadherin (1:1000, ab76011, Abcam), Snail (1:500, ab180714, Abcam), c-Myc (1:500, ab32072, Abcam), Cyclin D1 (1:2000, ab16663, Abcam), Survivin (1:1000, ab134170, Abcam), MET (1:1000, ab51067, Abcam), phosphorylated MET (1:1000, ab68141, Abcam), eIF4G1 (1:500, ab2609, Abcam), phosphorylated eIF4G1 (1:500, #2441, Cell signaling technology) and GAPDH (1:4000, ab8245, Abcam) at 4 °C overnight respectively. Second day, membranes were incubated with horseradish peroxidase (HRP)-conjugated secondary antibodies for 1 hour, which was visualized using enhanced chemiluminescence (ECL) substrates (Bio-Rad). Band intensity was quantified with ImageJ software. GAPDH was used as a normalization control.

**Statistical analysis**

Data from three independent assays was shown as mean ± standard deviation (SD). All data were in a normal distribution, and variance was similar between the groups that are being statistically compared. The correlation analysis among circTMTC1, miR-495 and MET in NPC patients were performed with Spearman’s Correlation. We used critical correlation coefficient for evaluating their correlation. If *r* > critical value for correlation coefficient, it is significant. With n = 32, the critical value is 0.4487 at α = 0.01 significance level. Kaplan-Meier analysis was used for assessing the overall survival rate of NPC patients. The variance of two groups was analyzed with the Student's t test. One-way analysis of variance (ANOVA) was used for comparisons of multiple groups. *P* < 0.05 was considered statistically significant. **P* < 0.05, ***P* < 0.01 and ****P* < 0.001.

**References**

1 Brennan B. Nasopharyngeal carcinoma. *Orphanet journal of rare diseases*. 2006; **1**: 23.

2 Wu L, Li C, Pan L. Nasopharyngeal carcinoma: A review of current updates. *Exp Ther Med*. 2018; **15**: 3687-3692.

3 Luo J, Wen Q, Li J, Xu L, Chu S, Wang W, et al. Increased expression of IRS-1 is associated with lymph node metastasis in nasopharyngeal carcinoma. *Int J Clin Exp Pathol*. 2014; **7**: 6117-6124.

4 Mahdavifar N, Towhidi F, Makhsosi BR, Pakzad R, Moini A, Ahmadi A, et al. Incidence and Mortality of Nasopharynx Cancer and Its Relationship With Human Development Index in the World in 2012. *World J Oncol*. 2016; **7**: 109-118.

5 Lee AW, Ma BB, Ng WT, Chan AT. Management of Nasopharyngeal Carcinoma: Current Practice and Future Perspective. *J Clin Oncol*. 2015; **33**: 3356-3364.

6 Zhang L, Chen QY, Liu H, Tang LQ, Mai HQ. Emerging treatment options for nasopharyngeal carcinoma. *Drug Des Devel Ther*. 2013; **7**: 37-52.

7 Chen YP, Wang ZX, Chen L, Liu X, Tang LL, Mao YP, et al. A Bayesian network meta-analysis comparing concurrent chemoradiotherapy followed by adjuvant chemotherapy, concurrent chemoradiotherapy alone and radiotherapy alone in patients with locoregionally advanced nasopharyngeal carcinoma. *Ann Oncol*. 2015; **26**: 205-211.

8 Kristensen LS, Andersen MS, Stagsted LVW, Ebbesen KK, Hansen TB, Kjems J. The biogenesis, biology and characterization of circular RNAs. *Nat Rev Genet*. 2019; **20**: 675-691.

9 Xie L, Mao M, Xiong K, Jiang B. Circular RNAs: A Novel Player in Development and Disease of the Central Nervous System. *Front Cell Neurosci*. 2017; **11**: 354.

10 Chen L, Zhang S, Wu J, Cui J, Zhong L, Zeng L, et al. circRNA_100290 plays a role in oral cancer by functioning as a sponge of the miR-29 family. *Oncogene*. 2017; **36**: 4551-4561.

11 Yu CY, Kuo HC. The emerging roles and functions of circular RNAs and their generation. *J Biomed Sci*. 2019; **26**: 29.

12 Yang J, Gong Y, Jiang Q, Liu L, Li S, Zhou Q, et al. Circular RNA Expression Profiles in Nasopharyngeal Carcinoma by Sequence Analysis. *Front Oncol*. 2020; **10**: 601.

13 Hong X, Liu N, Liang Y, He Q, Yang X, Lei Y, et al. Circular RNA CRIM1 functions as a ceRNA to promote nasopharyngeal carcinoma metastasis and docetaxel chemoresistance through upregulating FOXQ1. *Mol Cancer*. 2020; **19**: 33.

14 Wang J, Kong J, Nie Z, Chen D, Qiang J, Gao W, et al. Circular RNA Hsa_circ_0066755 as an Oncogene via sponging miR-651 and as a Promising Diagnostic Biomarker for Nasopharyngeal Carcinoma. *Int J Med Sci*. 2020; **17**: 1499-1507.

15 Ragan C, Goodall GJ, Shirokikh NE, Preiss T. Insights into the biogenesis and potential functions of exonic circular RNA. *Sci Rep*. 2019; **9**: 2048.

16 Zhou DN, Ye CS, Yang QQ, Deng YF. Integrated analysis of transcriptome profiling predicts potential lncRNA and circRNA targets in human nasopharyngeal carcinoma. *Oncol Lett*. 2020; **19**: 3123-3136.

17 Zou FW, Cao D, Tang YF, Shu L, Zuo Z, Zhang LY. Identification of CircRNA-miRNA-mRNA Regulatory Network in Gastrointestinal Stromal Tumor. *Front Genet*. 2020; **11**: 403.

18 Chen H, Wang X, Bai J, He A. Expression, regulation and function of miR-495 in healthy and tumor tissues. *Oncol Lett*. 2017; **13**: 2021-2026.

19 Lee SH, Jung YD, Choi YS, Lee YM. Targeting of RUNX3 by miR-130a and miR-495 cooperatively increases cell proliferation and tumor angiogenesis in gastric cancer cells. *Oncotarget*. 2015; **6**: 33269-33278.

20 Widodo, Djati MS, Rifa'i M. Role of MicroRNAs in carcinogenesis that potential for biomarker of endometrial cancer. *Ann Med Surg (Lond)*. 2016; **7**: 9-13.

21 Jiang X, Huang H, Li Z, He C, Li Y, Chen P, et al. MiR-495 is a tumor-suppressor microRNA down-regulated in MLL-rearranged leukemia. *Proc Natl Acad Sci U S A*. 2012; **109**: 19397-19402.

22 Feng X, Lv W, Wang S, He Q. miR495 enhances the efficacy of radiotherapy by targeting GRP78 to regulate EMT in nasopharyngeal carcinoma cells. *Oncol Rep*. 2018; **40**: 1223-1232.

23 Luraghi P, Schelter F, Kruger A, Boccaccio C. The MET Oncogene as a Therapeutical Target in Cancer Invasive Growth. *Front Pharmacol*. 2012; **3**: 164.

24 Zhang Y, Xia M, Jin K, Wang S, Wei H, Fan C, et al. Function of the c-Met receptor tyrosine kinase in carcinogenesis and associated therapeutic opportunities. *Mol Cancer*. 2018; **17**: 45.

25 Miekus K. The Met tyrosine kinase receptor as a therapeutic target and a potential cancer stem cell factor responsible for therapy resistance (Review). *Oncol Rep*. 2017; **37**: 647-656.

26 Gluck AA, Orlando E, Leiser D, Poliakova M, Nisa L, Quintin A, et al. Identification of a MET-eIF4G1 translational regulation axis that controls HIF-1alpha levels under hypoxia. *Oncogene*. 2018; **37**: 4181-4196.

27 Jeon HM, Lee J. MET: roles in epithelial-mesenchymal transition and cancer stemness. *Annals of translational medicine*. 2017; **5**: 5.

28 Ponzo MG, Lesurf R, Petkiewicz S, O'Malley FP, Pinnaduwage D, Andrulis IL, et al. Met induces mammary tumors with diverse histologies and is associated with poor outcome and human basal breast cancer. *Proc Natl Acad Sci U S A*. 2009; **106**: 12903-12908.

29 Qian CN, Guo X, Cao B, Kort EJ, Lee CC, Chen J, et al. Met protein expression level correlates with survival in patients with late-stage nasopharyngeal carcinoma. *Cancer Res*. 2002; **62**: 589-596.

30 Li YQ, Ren XY, He QM, Xu YF, Tang XR, Sun Y, et al. MiR-34c suppresses tumor growth and metastasis in nasopharyngeal carcinoma by targeting MET. *Cell Death Dis*. 2015; **6**: e1618.

31 Loh CY, Chai JY, Tang TF, Wong WF, Sethi G, Shanmugam MK, et al. The E-Cadherin and N-Cadherin Switch in Epithelial-to-Mesenchymal Transition: Signaling, Therapeutic Implications, and Challenges. *Cells*. 2019; **8**.

32 Wang Y, Shi J, Chai K, Ying X, Zhou BP. The Role of Snail in EMT and Tumorigenesis. *Current cancer drug targets*. 2013; **13**: 963-972.

33 Miller DM, Thomas SD, Islam A, Muench D, Sedoris K. c-Myc and cancer metabolism. *Clinical cancer research : an official journal of the American Association for Cancer Research*. 2012; **18**: 5546-5553.

34 Qie S, Diehl JA. Cyclin D1, cancer progression, and opportunities in cancer treatment. *Journal of molecular medicine*. 2016; **94**: 1313-1326.

35 Ito T, Shiraki K, Sugimoto K, Yamanaka T, Fujikawa K, Ito M, et al. Survivin promotes cell proliferation in human hepatocellular carcinoma. *Hepatology*. 2000; **31**: 1080-1085.

36 Dudekula DB, Panda AC, Grammatikakis I, De S, Abdelmohsen K, Gorospe M. CircInteractome: A web tool for exploring circular RNAs and their interacting proteins and microRNAs. *RNA Biol*. 2016; **13**: 34-42.

37 Yang JH, Li JH, Shao P, Zhou H, Chen YQ, Qu LH. starBase: a database for exploring microRNA-mRNA interaction maps from Argonaute CLIP-Seq and Degradome-Seq data. *Nucleic Acids Res*. 2011; **39**: D202-209.

38 Sekiyama N, Arthanari H, Papadopoulos E, Rodriguez-Mias RA, Wagner G, Leger-Abraham M. Molecular mechanism of the dual activity of 4EGI-1: Dissociating eIF4G from eIF4E but stabilizing the binding of unphosphorylated 4E-BP1. *Proc Natl Acad Sci U S A*. 2015; **112**: E4036-4045.

39 Dou C, Zhou Z, Xu Q, Liu Z, Zeng Y, Wang Y, et al. Hypoxia-induced TUFT1 promotes the growth and metastasis of hepatocellular carcinoma by activating the Ca(2+)/PI3K/AKT pathway. *Oncogene*. 2019; **38**: 1239-1255.

40 Chen YP, Chan ATC, Le QT, Blanchard P, Sun Y, Ma J. Nasopharyngeal carcinoma. *Lancet*. 2019; **394**: 64-80.

41 Wei F, Wu Y, Tang L, Xiong F, Guo C, Li X, et al. Trend analysis of cancer incidence and mortality in China. *Sci China Life Sci*. 2017; **60**: 1271-1275.

42 Lee AW, Ng WT, Chan LL, Hung WM, Chan CC, Sze HC, et al. Evolution of treatment for nasopharyngeal cancer--success and setback in the intensity-modulated radiotherapy era. *Radiother Oncol*. 2014; **110**: 377-384.

43 Yin L, Chen J, Ma C, Pei S, Du M, Zhang Y, et al. Hsa_circ_0046263 functions as a ceRNA to promote nasopharyngeal carcinoma progression by upregulating IGFBP3. *Cell Death Dis*. 2020; **11**: 562.

44 Liu R, Zhou M, Zhang P, Zhao Y, Zhang Y. Cell proliferation and invasion is promoted by circSERPINA3 in nasopharyngeal carcinoma by regulating miR-944/MDM2 axis. *J Cancer*. 2020; **11**: 3910-3918.

45 Han TS, Hur K, Cho HS, Ban HS. Epigenetic Associations between lncRNA/circRNA and miRNA in Hepatocellular Carcinoma. *Cancers (Basel)*. 2020; **12**.

46 Yang F, Chen Y, Xue Z, Lv Y, Shen L, Li K, et al. High-Throughput Sequencing and Exploration of the lncRNA-circRNA-miRNA-mRNA Network in Type 2 Diabetes Mellitus. *Biomed Res Int*. 2020; **2020**: 8162524.

47 Gong J, Jiang H, Shu C, Hu MQ, Huang Y, Liu Q, et al. Integrated analysis of circular RNA-associated ceRNA network in cervical cancer: Observational Study. *Medicine (Baltimore)*. 2019; **98**: e16922.

48 Li W, Lu H, Wang H, Ning X, Liu Q, Zhang H, et al. Circular RNA TGFBR2 acts as a ceRNA to suppress nasopharyngeal carcinoma progression by sponging miR-107. *Cancer letters*. 2021; **499**: 301-313.

49 Zhu L, Liu Y, Yang Y, Mao XM, Yin ZD. CircRNA ZNF609 promotes growth and metastasis of nasopharyngeal carcinoma by competing with microRNA-150-5p. *European review for medical and pharmacological sciences*. 2019; **23**: 2817-2826.

50 Yan L, Yao J, Qiu J. miRNA-495 suppresses proliferation and migration of colorectal cancer cells by targeting FAM83D. *Biomedicine & pharmacotherapy = Biomedecine & pharmacotherapie*. 2017; **96**: 974-981.

51 Liu C, Jian M, Qi H, Mao WZ. MicroRNA 495 Inhibits Proliferation and Metastasis and Promotes Apoptosis by Targeting Twist1 in Gastric Cancer Cells. *Oncology research*. 2019; **27**: 389-397.

52 Eun JW, Kim HS, Shen Q, Yang HD, Kim SY, Yoon JH, et al. MicroRNA-495-3p functions as a tumor suppressor by regulating multiple epigenetic modifiers in gastric carcinogenesis. *J Pathol*. 2018; **244**: 107-119.

53 Bai Z, Wang J, Wang T, Li Y, Zhao X, Wu G, et al. The MiR-495/Annexin A3/P53 Axis Inhibits the Invasion and EMT of Colorectal Cancer Cells. *Cell Physiol Biochem*. 2017; **44**: 1882-1895.

54 Lytle JR, Yario TA, Steitz JA. Target mRNAs are repressed as efficiently by microRNA-binding sites in the 5' UTR as in the 3' UTR. *Proc Natl Acad Sci U S A*. 2007; **104**: 9667-9672.

55 You X, Zhou Z, Chen W, Wei X, Zhou H, Luo W. MicroRNA-495 confers inhibitory effects on cancer stem cells in oral squamous cell carcinoma through the HOXC6-mediated TGF-beta signaling pathway. *Stem cell research & therapy*. 2020; **11**: 117.

56 Gherardi E, Birchmeier W, Birchmeier C, Vande Woude G. Targeting MET in cancer: rationale and progress. *Nat Rev Cancer*. 2012; **12**: 89-103.

57 Li Y, Li W, He Q, Xu Y, Ren X, Tang X, et al. Prognostic value of MET protein overexpression and gene amplification in locoregionally advanced nasopharyngeal carcinoma. *Oncotarget*. 2015; **6**: 13309-13319.

58 Ma PC, Tretiakova MS, MacKinnon AC, Ramnath N, Johnson C, Dietrich S, et al. Expression and mutational analysis of MET in human solid cancers. *Genes, chromosomes & cancer*. 2008; **47**: 1025-1037.

59 Faoro L, Cervantes GM, El-Hashani E, Salgia R. MET receptor tyrosine kinase. *Journal of thoracic oncology : official publication of the International Association for the Study of Lung Cancer*. 2009; **4**: S1064-1065.

60 Jackson RJ, Hellen CU, Pestova TV. The mechanism of eukaryotic translation initiation and principles of its regulation. *Nat Rev Mol Cell Biol*. 2010; **11**: 113-127.

61 Jaiswal PK, Koul S, Palanisamy N, Koul HK. Eukaryotic Translation Initiation Factor 4 Gamma 1 (EIF4G1): a target for cancer therapeutic intervention? *Cancer Cell Int*. 2019; **19**: 224.

62 Han Y, Luo Y, Zhao J, Li M, Jiang Y. Overexpression of c-Met increases the tumor invasion of human prostate LNCaP cancer cells in vitro and in vivo. *Oncol Lett*. 2014; **8**: 1618-1624.

**Acknowledgements**

We would like to give our sincere gratitude to the reviewers for their constructive comments.

**Conflict of Interest Statement**

The authors declare that there is no conflict of interest.

**Ethics Statement**

This study got approval from the Ethics Committee of the Xiangya Hospital of Central South University. All patients provided written informed consent. Animal experiments were approved by the Animal Care and Use Committee of the Xiangya Hospital of Central South University.

**Author Contribution Statement**

YJZ: Conceptualization; Funding acquisition; Writing-original draft;

CL: Data curation; Resources;

YZ: Methodology; Formal analysis; Investigation; Software; Visualization;

ZZL: Project administration; Supervision; Validation; Writing-review & editing.

All authors have read and approved the final version of this manuscript to be published.

**Funding Statement**

This work was supported by China Scholarship Council (ZYJ: NO. 201906375015), the Science Foundation of Xiangya Hospital for Young Scholar (LZZ: NO. 2018Q012), National Natural Science Foundation of China (LZZ: No. 82003239) and (ZYJ: 82002887).

**Declarations**

**Consent for Publication**

The informed consent was obtained from study participants.

**Availability of Data and Material**

All data generated or analyzed during this study are included in this article. The datasets used and/or analyzed during the current study are available from the corresponding author on reasonable request.

**Tables**

**Table 1. The primers for qRT-PCR used in this study.**

| Genes | Primer sequences (5'-3') |
| --- | --- |
| circTMTC1 | F: 5’-CAGAACCCAAGAGCAGTGGA-3’ |
|  | R: 5’-ACAGCAGACACGCTAACACG-3’ |
| miR-495 | F: 5’-GGCGAAACAAACATGGTGCA-3’ |
|  | R: 5’-GTCGTATCCAGTGCAGGGTCCGAGGTA  TTCGCACTGGATACGAC AAGAAG-3’ |
| TMTC1 | F: 5’-GCTGTTTCTATTGGCCTTTCTC-3’ |
|  | R: 5’-TGTCTCTTTCACCAGCATCG-3’ |
| MET | F: 5’-CTGGGCACCGAAAGATAAACC-3’ |
|  | R: 5’-GTGTTTCCGCGGTGAAGTTG-3’ |
| GAPDH | F: 5’-CCAGGTGGTCTCCTCTGA3’ |
|  | R: 5’-GCTGTAGCCAAATCGTTGT-3’ |
| U6 | F: 5’-CTCGCTTCGGCAGCACA-3’ |
|  | R: 5’-AACGCTTCACGAATTTGCGT-3’ |

**Figure Legends**

**Figure 1. Abnormal expression of circTMTC1 and miR-495 was associated with the poor prognosis of NPC.** (A) Genomic loci of circTMTC1. Sanger sequencing was conducted to validate the back-splicing junction. (B) The relative expression of TMTC1 and circTMTC1 in 5-8F cells after RNase R treatment at 2 U/μg. Data from three independent experiments was normalized to GAPDH. (C) The relative expression of TMTC1 and circTMTC1 in 5-8F cells treated with actinomycin D for indicated time. Data from three independent experiments was normalized to GAPDH. (D) The relative expression of GAPDH, U6 snRNA and circTMTC1 in the cytoplasm and nucleus of 5-8F cells. (E) FISH for circTMTC1 (green) in 5-8F cells. The nuclei were stained with DAPI (blue). (F) The relative expression of circTMTC1 in tumor and normal nasopharyngeal epithelium tissues from NPC patients (tumor tissue, n=32; normal nasopharyngeal epithelium tissue, n=16). Data was normalized to GAPDH. (G) The survival variance between circTMTC1^high^ (n=16) and circTMTC1^low^ (n=16) patients was evaluated using the Kaplan-Meier curve. (H) miR-495 expression in tumor and normal nasopharyngeal epithelium tissues from NPC patients (tumor tissue, n=32; normal nasopharyngeal epithelium tissue, n=16). Data was normalized to U6 snRNA. (I) The survival variance between miR-495^high^ (n=16) and miR-495^low^ (n=16) patients was evaluated using the Kaplan-Meier curve. The expression of circTMTC1 (J) and miR-495 (K) in NP69 and NPC cells. * *P* < 0.05, ** *P* < 0.01 and *** *P* < 0.001.

**Figure 2. CircTMTC1 knockdown and miR-495 overexpression suppressed NPC malignant phenotypes and enhanced cell apoptosis.** 5-8F and SUNE1 cells were transfected with sh-NC, sh-circTMTC1, mimics NC or miR-495 mimics respectively. (A&B) The relative expression of circTMTC1 and miR-495. Data from three independent experiments was normalized to GAPDH or U6 snRNA respectively. (C&D) Colony formation analysis of NPC cells. (E&F) Cell apoptosis analysis by flow cytometry. Scratch wound healing (G&H) and transwell invasion (I&J) analysis for 5-8F and SUNE1 cells. (K&L) The expression of E/N-cadherin, Snail, c-Myc, Cyclin D1 and Survivin. * *P* < 0.05, ** *P* < 0.01 and *** *P* < 0.001.

**Figure 3. CircTMTC1 directly bound to miR-495 to reduce its abundance in NPC.** (A) Correlation analysis of the expression of circTMTC1 and miR-495 in NPC patients (n=32). (B) miR-495 expression in 5-8F and SUNE1 cells transfected with sh-NC or sh-circTMTC1. Data from three independent experiments was normalized to GAPDH. (C) Predicted binding site of miR-495 in circTMTC1. (D) The relative luciferase activity of circTMTC1 wild type or mutated reporter in NPC cells. (E) Enrichment of circTMTC1 and miR-495 in anti-Ago2 or normal IgG-immunoprecipitated fractions. (F) RNA pull-down assays for evaluating the interaction between circTMTC1 and miR-495. (G) FISH for circTMTC1 (green) and miR-495 (red) in NPC cells. The nuclei were stained with DAPI (blue). * *P* < 0.05, ** *P* < 0.01 and *** *P* < 0.001.

**Figure 4. CircTMTC1 promoted NPC cell malignant phenotypes and enhanced cell apoptosis by directly targeting miR-495.** NPC cells were transfected with vector control, circTMTC1, circTMTC1+mimics NC or circTMTC1+miR-495 mimics respectively. (A) The relative expression of circTMTC1. Data from three independent experiments was normalized to GAPDH. (B&C) Colony formation analysis of NPC cells. (D&E) Cell apoptosis analysis by flow cytometry. Scratch wound healing (F&G) and transwell invasion (H&I) analysis for NPC cells. (J&K) The expression of E/N-cadherin, Snail, c-Myc, Cyclin D1 and Survivin. * *P* < 0.05, ** *P* < 0.01 and *** *P* < 0.001.

**Figure 5. miR-495 directly targeted MET to inhibit the MET-eIF4G1 translational regulation axis.** (A) The relative expression of MET in tumor and normal nasopharyngeal epithelium tissues from NPC patients (tumor tissue, n=32; normal nasopharyngeal epithelium tissue, n=16). (B) The survival variance between MET^high^ (n=16) and MET^low^ (n=16) patients was evaluated using the Kaplan-Meier curve. (C) Correlation analysis of the expression of circTMTC1 and MET in NPC patients (n=32). (D) Correlation analysis of the expression of MET and miR-495 in NPC patients (n=32). (E) Predicted binding site for miR-495 in MET. (F) The luciferase activity of MET wild type or mutated reporter in NPC cells. (G) The expression of MET in 5-8F and SUNE1 cells transfected with sh-NC, sh-circTMTC1, mimics NC or miR-495 mimics. (H&I) Western blotting analysis of MET, phosphorylated MET, eIF4G1 and phosphorylated eIF4G1. * *P* < 0.05, ** *P* < 0.01 and *** *P* < 0.001.

**Figure 6. miR-495 knockdown promoted NPC malignant phenotypes and inhibited cell apoptosis by activating the MET-eIF4G1 translational regulation pathway.** NPC cells were transfected with inhibitor NC, miR-495 inhibitor, miR-495 inhibitor+sh-NC or miR-495 inhibitor+sh-MET respectively or treated with 4EGI-1 after miR-495 inhibitor transfection. (A&B) The relative expression of miR-495 and MET. Data from three independent experiments was normalized to GAPDH. (C) Western blotting analysis of MET. (D&E) Colony formation analysis of NPC cells. (F&G) Cell apoptosis analysis by flow cytometry. Scratch wound healing (H&I) and transwell invasion (J&K) analysis for 5-8F and SUNE1 cells. * *P* < 0.05, ** *P* < 0.01 and *** *P* < 0.001.

**Figure 7. miR-495 knockdown promoted EMT and cell proliferation-related signal by activating the MET-eIF4G1 translational regulation pathway.** NPC cells were transfected with inhibitor NC, miR-495 inhibitor, miR-495 inhibitor+sh-NC or miR-495 inhibitor+sh-MET respectively or treated with 4EGI-1 after miR-495 inhibitor transfection. (A&B) Western blotting analysis of E/N-cadherin, Snail, c-Myc, Cyclin D1 and Survivin in 5-8F and SUNE1 cells. GAPDH was used as a loading control. * *P* < 0.05, ** *P* < 0.01 and *** *P* < 0.001.

**Figure 8. CircTMTC1 silence suppressed NPC growth and lung metastasis through targeting the miR-495-MET-eIF4G1 axis *in vivo*.** (A) Photos of excised tumors from subcutaneous xenograft mice. (B) Tumor volume was monitored every four days. (C) Weight of excised tumors. (D) The abundance of circTMTC1, miR-495 and MET mRNA by qRT-PCR in tumor tissues from subcutaneous xenograft mice. (E) IHC staining of Ki-67, MET and eIF4G1 in tumor sections from subcutaneous xenograft mice. The result was representative of three independent experiments. (F&G) Western blotting analysis of MET, phosphorylated MET, eIF4G1, phosphorylated eIF4G1, E/N-cadherin, Snail, c-Myc, Cyclin D1 and Survivin. (H) Photos of excised lungs from intravenous xenograft mice. (I) Number of tumor nodule in the lungs. (J) H&E staining of lung sections from intravenous xenograft mice. * *P* < 0.05, ** *P* < 0.01 and *** *P* < 0.001.
