# Supplementary figures and images for: CircTMTC1 contributes to nasopharyngeal carcinoma progression through targeting miR-495-MET-eIF4G1 translational regulation axis
Source: Cell Death Dis. 2022 Mar 18;13(3):250. doi: 10.1038/s41419-022-04686-z (PMC8930977; doi:10.1038/s41419-022-04686-z)

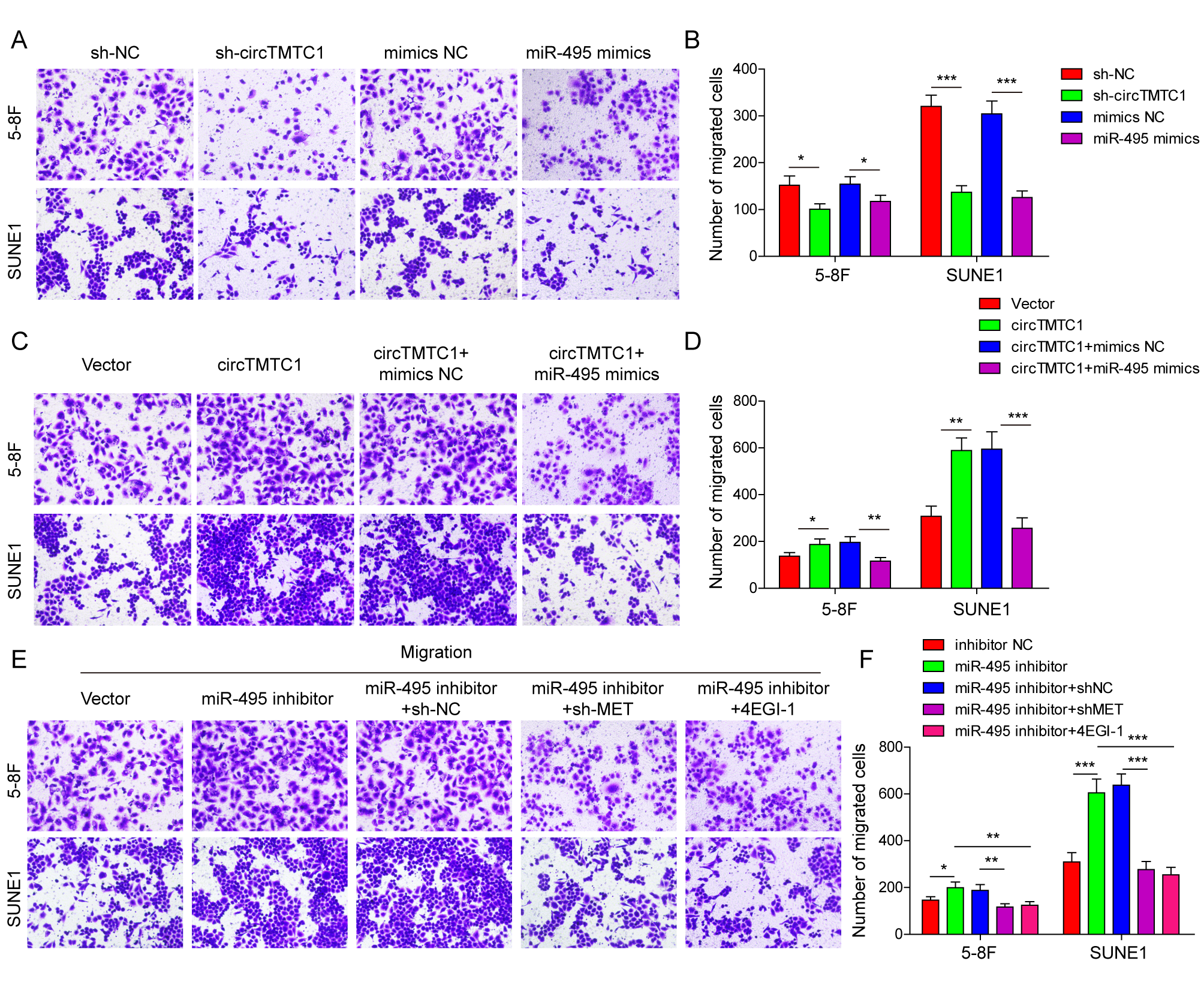

Supplement: Supplementary file 2 — Supplementary Figure 1 [file 41419_2022_4686_MOESM2_ESM.tif]

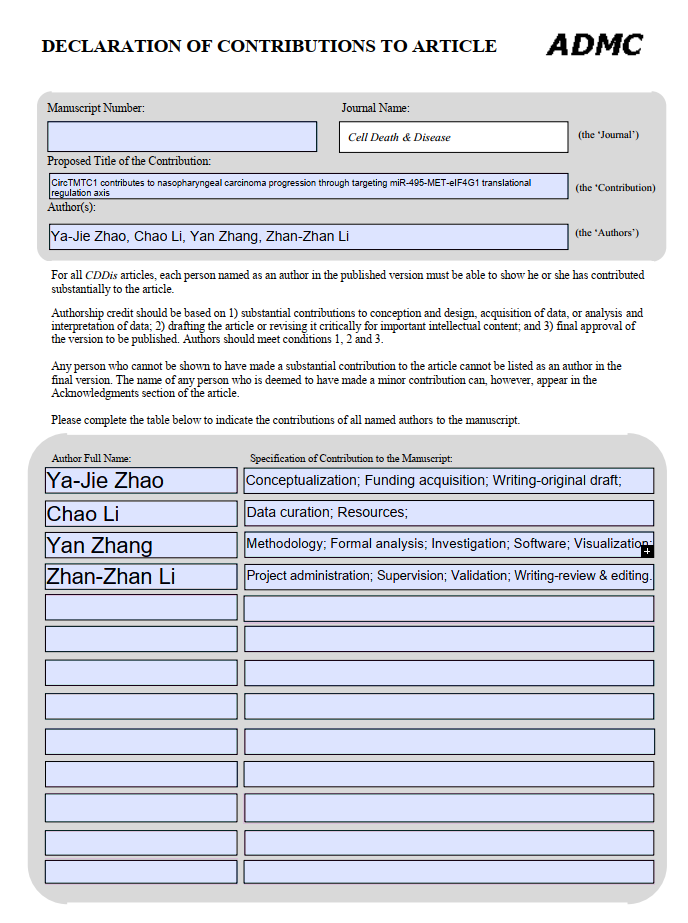


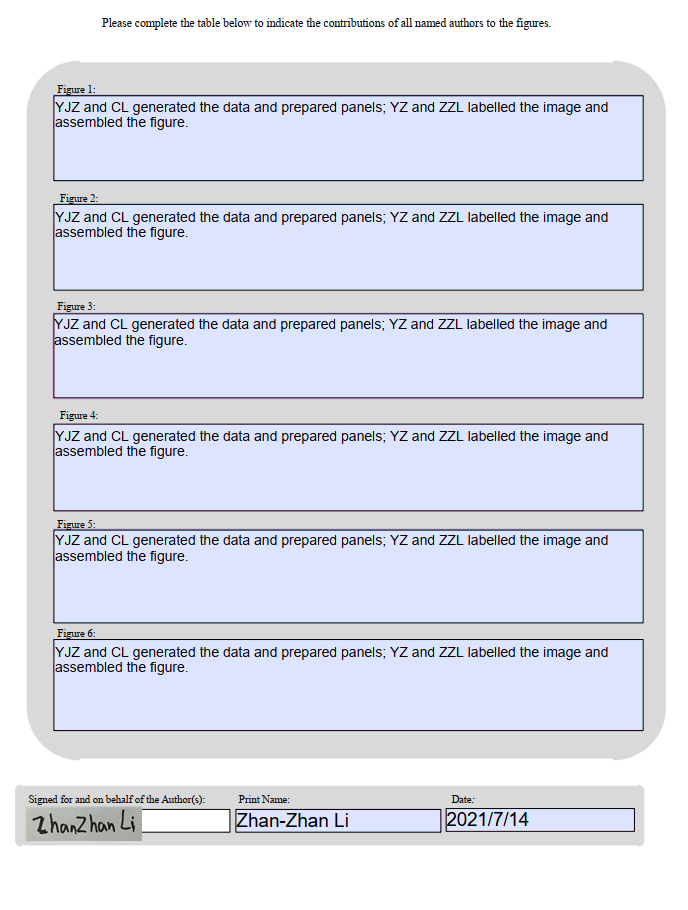

Supplement: Supplementary file 4 — author contribution form [file 41419_2022_4686_MOESM4_ESM.docx]
