# Supplementary material for: CircTMTC1 contributes to nasopharyngeal carcinoma progression through targeting miR-495-MET-eIF4G1 translational regulation axis
Source: Cell Death Dis. 2022 Mar 18;13(3):250. doi: 10.1038/s41419-022-04686-z (PMC8930977; doi:10.1038/s41419-022-04686-z)
Supplement: Supplementary file 3 — Supplementary Figure Legends [file 41419_2022_4686_MOESM3_ESM.doc]

**Supplementary Figure 1. 5-8F and SUNE1 cell migration were analyzed by transwell migration assays for 5-8F and SUNE1 cells.** (A&B) Transwell migration analysis for 5-8F and SUNE1 cells transfected with sh-NC, sh-circTMTC1, mimics NC or miR-495 mimics. (C&D) Transwell migration analysis for 5-8F and SUNE1 cellstransfected with vector control, circTMTC1, circTMTC1+mimics NC or circTMTC1+miR-495 mimics. (E&F) Transwell migration analysis for 5-8F and SUNE1 cells transfected with inhibitor NC, miR-495 inhibitor, miR-495 inhibitor+sh-NC or miR-495 inhibitor+sh-MET.
